# Supplementary material for: Hybrid passive micromixer using combined traditional microfabrication and 3D printing for gold nanoparticle synthesis
Source: PLoS One. 2026 Feb 18;21(2):e0342823. doi: 10.1371/journal.pone.0342823 (PMC12915908; doi:10.1371/journal.pone.0342823)
Supplement: S1 File — (DOCX) [file pone.0342823.s001.docx]

***Supplementary Material***

**Hybrid Passive Micromixer Using Combined Traditional Microfabrication and 3D Printing for Nanoparticle Synthesis**

Yasser Aldaghestani^1,2^, Andreas Schiffer^1,3*^, Anas Alazzam^1,2*^

^1^ Department of Mechanical and Nuclear Engineering, Khalifa University of Science and Technology, Abu Dhabi, 127788, United Arab Emirates

^2^ System on Chip Lab, Khalifa University of Science and Technology, 127788, Abu Dhabi, United Arab Emirates

^3^ Advanced Research and Innovation Center (ARIC), Khalifa University of Science and Technology, Abu Dhabi, 127788, United Arab Emirates

* Corresponding authors. [andreas.schiffer@ku.ac.ae](mailto:andreas.schiffer@ku.ac.ae); [anas.alazzam@ku.ac.ae](mailto:anas.alazzam@ku.ac.ae)

**1. Mesh independence study**

Selecting an appropriate mesh size is essential to balance numerical accuracy and computational cost. Accordingly, a mesh-sensitivity study was conducted for the hybrid micromixer prior to the production simulations. Mesh dependence was assessed at Re= 1 using the mixing index evaluated at a downstream location of x= 6mm. As shown in Fig. 3, the mixing index decreased monotonically with mesh refinement and gradually approached a plateau. For the three finest meshes 5.0, 7.0, and 8.0 million elements, the corresponding mixing indices were 0.325, 0.315, and 0.310, respectively. The change between the two coarser resolutions was 3.03%, whereas further refinement resulted in a smaller variation of 1.70%. Since additional refinement produced less than a 2% change in the monitored metric and did not alter the qualitative flow or concentration fields, the intermediate mesh resolution was adopted for all simulations.

The computational domain was discretized using quadratic tetrahedral elements with a minimum element size of 0.5 µm. As illustrated in Fig S1, mesh refinement was focused on regions where the helical structure approaches the channel walls to accurately resolve steep velocity and concentration gradients within the narrow gaps and capture the resulting complex flow features.


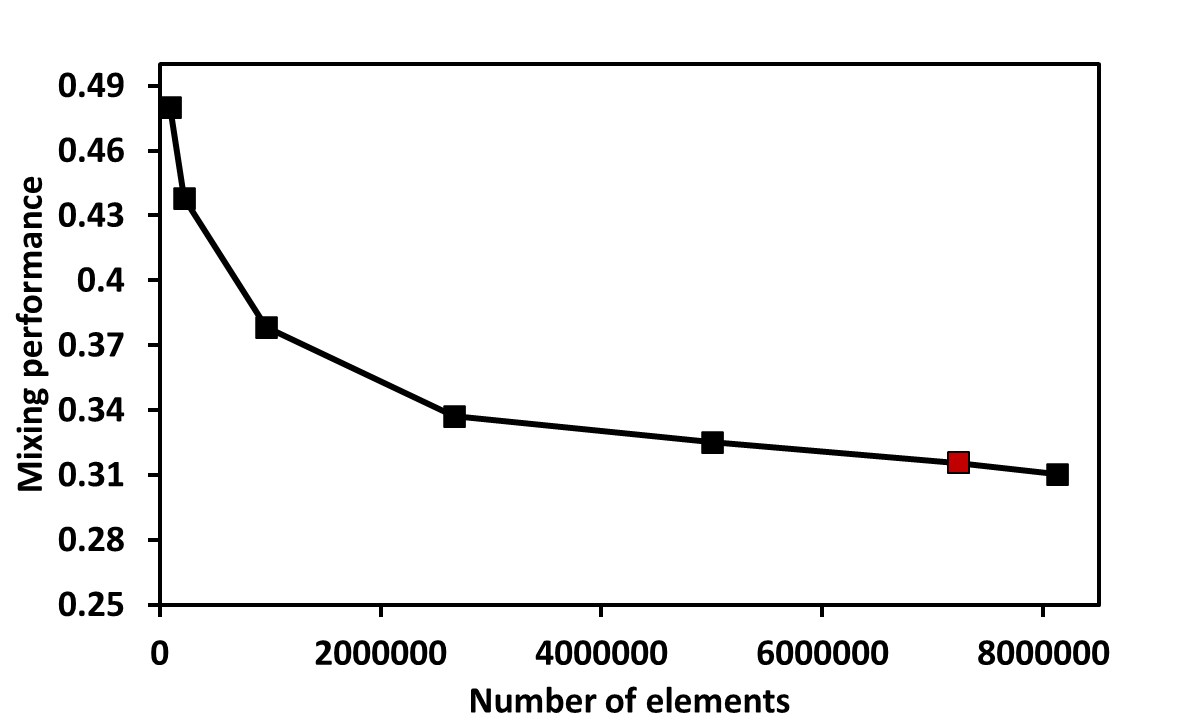


**Fig S1.** **Mesh independence test.** mixing performance at fixed location (x= 6 mm) for seven mesh resolutions.

**2. Numerical Model Validation**

This section focuses on model validation by replicating well-known geometries in existing literature. Specifically, three distinct mixer designs were simulated and are illustrated in Fig S2: the T-mixer [1], the Tesla model [2], and the caterpillar design [3], all of which are recognized for their reported mixing efficiency. These designs were selected to enable a comparative analysis of different mixing mechanisms within microchannels of uniform dimensions (20 mm in length, 500 µm in width, 400 µm in height). For example, the Tesla-like mixer operates on the principle of flow redirection to induce collisions between fluid streamlines, as detailed by Hong et al. [2]. In contrast, the caterpillar mixer employs a split-and-recombine technique, effectively creating multiple fluid layers and thereby reducing the diffusion path for particles, as discussed in [4, 5].

An analysis of the mixing performance across varying Reynolds numbers (3.7, 18.2, 37.04) reveals distinct design behaviors, the T-mixer's performance declines with increasing Reynolds number, significantly underperforming compared to the other models. This behavior is attributed to the T-mixer's reliance on diffusive mixing, which becomes less effective at higher flow rates. As the Reynolds number increases, convective transport dominates, reducing the fluid's residence time within the mixing region and limiting molecular diffusion. In contrast, the Tesla-like and Caterpillar mixers utilize chaotic advection and streamline collisions, thereby enhancing mixing efficiency as the flow velocity increases. The effectiveness of the Tesla-like mixer improves with higher flow rates due to stronger fluid collisions. In contrast, the Caterpillar mixer similarly benefits from enhanced streamlines interactions, making diffusion-driven mixing less critical.

A comparison is made between the numerical predictions in this work and those reported in the established literature [3]. As shown in Fig S2, there is strong agreement between the numerical simulations and the referenced published works across various Reynolds numbers. The average discrepancies, calculated at the end of the channels (20 mm), are 4.6% for the T-junction design, 3.9% for the Tesla model, and 1.4% for the Caterpillar design. This close agreement between the predictions of the current numerical model and the established benchmarks highlights the accuracy of the simulation approach, confirming its reliability in predicting the mixing performance.


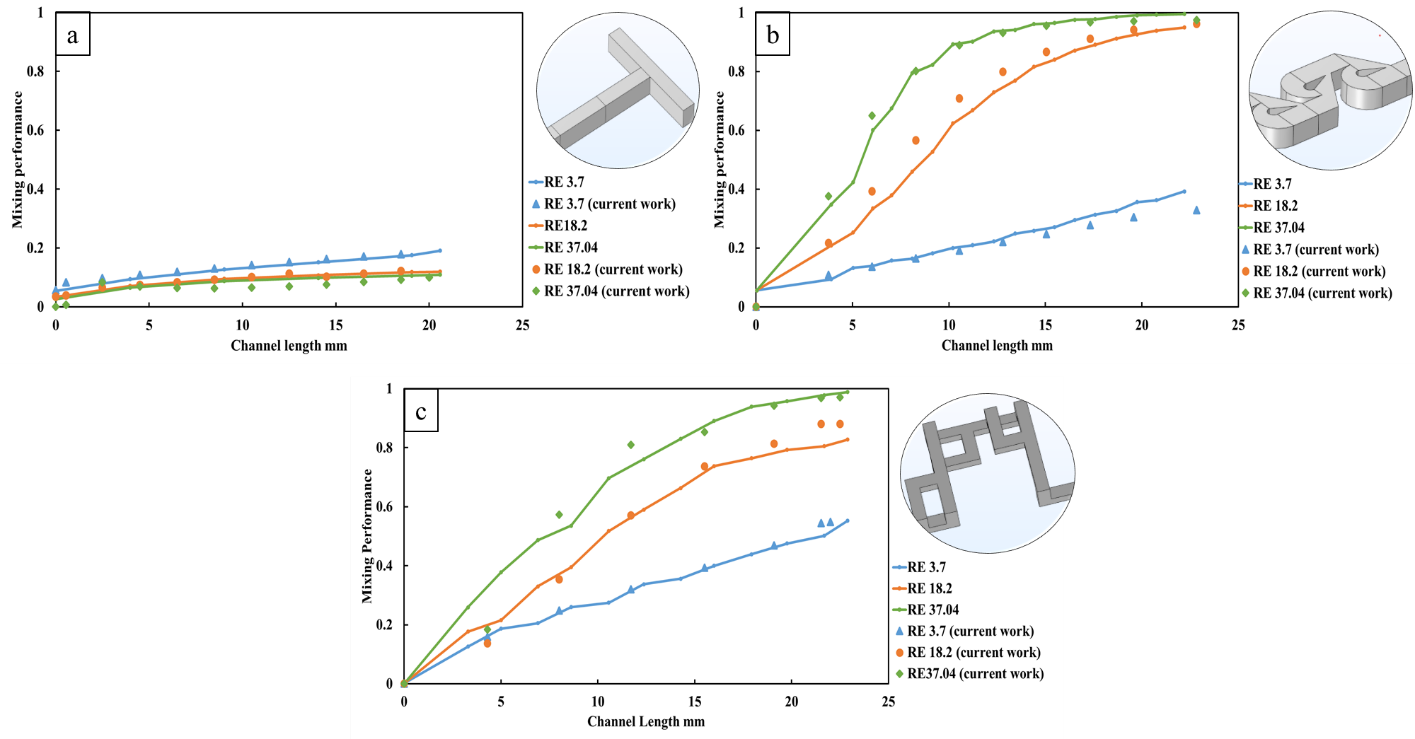


**Fig S2.** Comparative analysis of mixing performance: across (a) T-mixer, (b) Tesla-like, (c) Caterpillar, demonstrating the alignment of our simulation results with published literature [3].

**3. Comparison of the mixing performance of passive micromixers reported in the literature**

**Table S1.** Comparison of mixing efficiencies for various passive micromixer designs at Reynolds numbers of 0.01 and 1, evaluated at a channel length of 5 mm.

| **Reference** | **Micromixer design** | **Mixing mechanism** | **Mixing index at Re=0.01** | **Mixing index at Re=1** |
| --- | --- | --- | --- | --- |
| Hossain et al. [6] | Modified Tesla micromixer | Inertial force, SAR | 0.465 | 0.203 |
| Hossain et al. [7] | Curved micromixer | Inertial force (secondary  flow, Dean vortex) | 0.561 | 0.224 |
| Alam et al. [8] | Curved micromixer with grooves | Inertial force (secondary  flow, Dean vortex) | 0.554 | 0.221 |
| Li et al. [9] | Micromixer with dislocation  sub-channels | Inertial force, SAR | 0.268 | 0.125 |
| Ansari et al. [10] | 3D serpentine micromixer | Inertial force, chaotic mixing,  multi-lamination | 0.546 | 0.377 |
| Hossain et al. [11] | 3D serpentine SAR micromixer | Inertial force, chaotic mixing,  multi-lamination | 0.649 | 0.472 |
| Park et al. [12] | Improved serpentine laminating micromixer | Inertial force, chaotic mixing,  multi-lamination | 0.904 | 0.537 |
| Kim et al. [13] | Barrier-embedded chaotic  micromixer | Inertial force, chaotic mixing | 0.310 | 0.226 |
| Juraeva et al. [14] | Planar multiple-baffle micromixer with alternating submergence scheme | Secondary vortices, chaotic mixing | - | 0.570 |
| Neves et al. [15] | Side-feeds conical-entrance micromixer | chaotic mixing | 0.8 | - |
| Current work | Hybrid micromixer | Inertial force, chaotic mixing | 0.99 | 0.731 |

**4. Y-shaped mixing performance**

This section presents observations from microfluidic experiments conducted on a typical Y-junction micromixer without the 3D-printed helix. Fig S3 shows the device and the flow at the three specified rates captured during the experiments. The figure shows that the Y-junction micromixer exhibited a lack of mixing between the two fluid streams, reporting a mixing performance of only around 20%. This outcome was anticipated, as the mixing in such a device relies heavily on diffusion, a slow process, and the interface where the two streams meet is relatively small. This limited contact area is insufficient for effective mixing at the applied flow rates, as diffusion alone cannot achieve the rapid homogenization of the fluid.


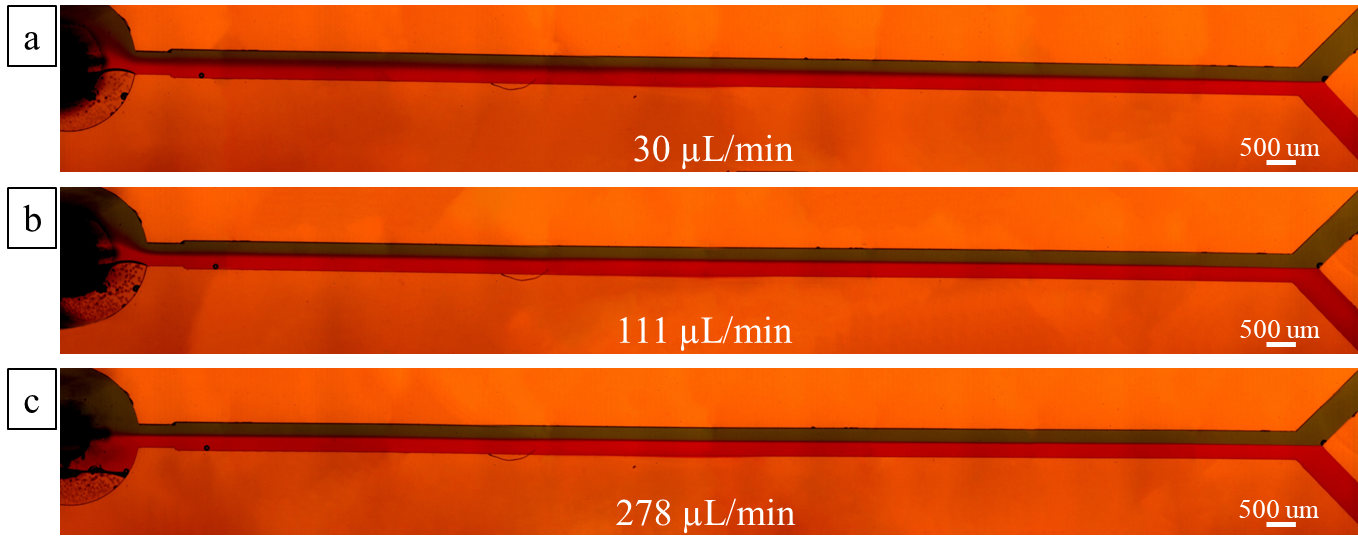


**Fig S3.** **Images captured during the microfluidic experiments performed at various flow rates.** (a) 30 µL/min, (b) 111 µL/min, and (c) 278 µL/min.

**References**

1. Wang, W., et al., *Visualization of micro-scale mixing in miscible liquids using μ-LIF technique and drug nano-particle preparation in T-shaped micro-channels.* Chemical Engineering Journal, 2012. **192**: p. 252-261.

2. Hong, C.C., J.W. Choi, and C.H. Ahn, *A novel in-plane passive microfluidic mixer with modified Tesla structures.* Lab Chip, 2004. **4**(2): p. 109-13.

3. Enders, A., et al., *3D Printed Microfluidic Mixers-A Comparative Study on Mixing Unit Performances.* Small, 2019. **15**(2): p. e1804326.

4. Norbert, S., F. Thomas, and W. Helmut, *A modular microfluid system with an integrated micromixer.* Journal of Micromechanics and Microengineering, 1996. **6**(1): p. 99.

5. Rajabi, N., et al., *A Chaotic Advection Enhanced Microfluidic Split-and-Recombine Mixer for the Preparation of Chemical and Biological Probes.* JOURNAL OF CHEMICAL ENGINEERING OF JAPAN, 2012. **45**(9): p. 703-707.

6. Hossain, S., et al., *Analysis and optimization of a micromixer with a modified Tesla structure.* Chemical Engineering Journal, 2010. **158**(2): p. 305-314.

7. Hossain, S., M. Ansari, and K.-Y. Kim, *Evaluation of the mixing performance of three passive micromixers.* Chemical Engineering Journal, 2009. **150**(2-3): p. 492-501.

8. Alam, A. and K.-Y. Kim, *Analysis of mixing in a curved microchannel with rectangular grooves.* Chemical Engineering Journal, 2012. **181**: p. 708-716.

9. Li, J., G. Xia, and Y. Li, *Numerical and experimental analyses of planar asymmetric split‐and‐recombine micromixer with dislocation sub‐channels.* Journal of Chemical Technology & Biotechnology, 2013. **88**(9): p. 1757-1765.

10. Ansari, M.A., *Parametric study on mixing of two fluids in a three-dimensional serpentine microchannel.* Chemical Engineering Journal, 2009. **146**(3): p. 439-448.

11. Hossain, S. and K.-Y. Kim, *Mixing analysis in a three-dimensional serpentine split-and-recombine micromixer.* Chemical Engineering Research and Design, 2015. **100**: p. 95-103.

12. Park, J.M., et al., *Improved serpentine laminating micromixer with enhanced local advection.* Microfluidics and Nanofluidics, 2008. **4**: p. 513-523.

13. Kim, D.S., et al., *A barrier embedded chaotic micromixer.* Journal of micromechanics and microengineering, 2004. **14**(6): p. 798.

14. Juraeva, M. and D.-J. Kang, *Mixing Performance of a Passive Micromixer Based on Multiple Baffles and Submergence Scheme.* Micromachines, 2023. **14**(5): p. 1078.

15. Neves, R.V., et al., *Evaluation of the mixing index in a micromixer of side feeds in a conical chamber.* Chemical Engineering and Processing - Process Intensification, 2024. **200**: p. 109771.
